# Diffuse interstitial fibrosis in well-controlled hypertension

**DOI:** 10.1186/1532-429X-15-S1-P251

**Published:** 2013-01-30

**Authors:** Thomas A Treibel, Filip Zemrak, Steven K White, Daniel Sado, Sanjay M Banypersad, Viviana Maestrini, Mark Caulfield, Steffen E Petersen, James Moon

**Affiliations:** 1 The Heart Hospital Imaging Centre, University College London, London, UK; 2 The Hatter Cardiovascular Institute, University College London Hospitals NHS Trust, London, UK; 3 Cardiovascular Biomedical Research Unit, Barts and the London School of Medicine and Dentistry, Queen Mary University of London, London, UK

## Background

Diffuse myocardial fibrosis (DMF) is an important factor in cardiac disease but until recently could only be accurately assessed with biopsy. We hypothesised that DMF measured by EQ-CMR is elevated in isolated systemic hypertension. As such DMF may be a key biomarker in assessing the cardiac effects of systemic hypertension.

## Methods

ECV measurement was by EQ-CMR. The T1 mapping sequence was ShMOLLI. The contrast agent was Gadoterate meglumine (Dotarem) at 0.1mmol/Kg (bolus) plus infusion at 15 minutes at 0.0011 mmol/kg/min. CMR was at 1.5T (Siemens Avanto).

ECV was measured in 43 well-controlled hypertensive patients from a specialist tertiary centre (median age 56, range 21 to 78, 55% male) and 50 healthy volunteers (median age 47, range 28 to 69, 58% male).

ECV was calculated by ECV = (1-hematocrit) x (1/T1)myo ÷ (1/T1)blood.

## Results

**Figure 1:**
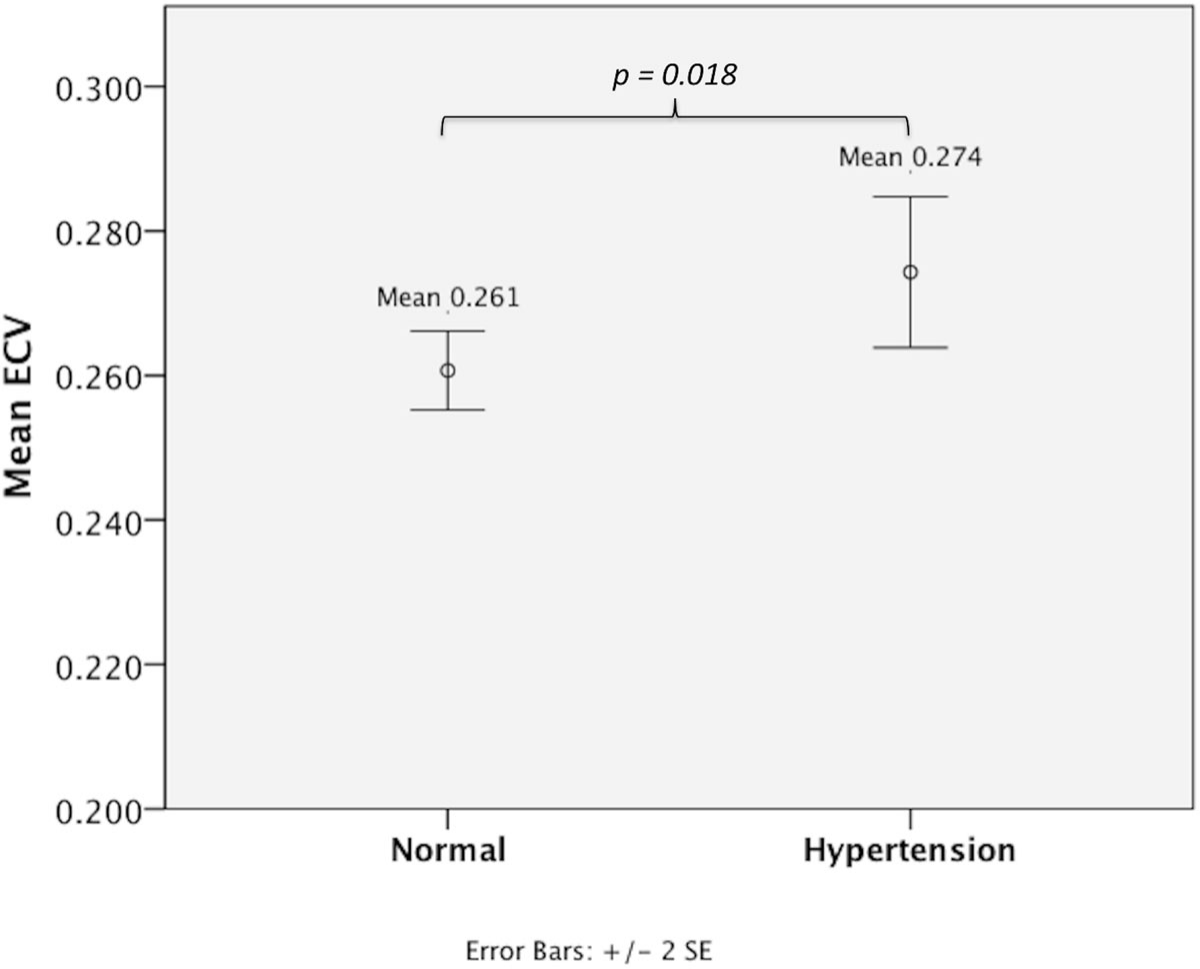
ECV measurements by EQ-CMR showed significantly higher ECV in 43 hypertensives than 50 gender-matched, normotensive controls (0.274 versus 0.261, *p*=*0.018*).

The 43 hypertensive subjects had a significantly higher ECV than the 50 gender-matched normotensive controls (0.274 versus 0.261, *p*=*0.018*; Figure 1). Although the mean mass index (85.0g/m^2^ versus 62.7g/m^2^) was significantly higher in hypertensive subjects, there was no correlation between ECV and mass index.

**Figure 2:**
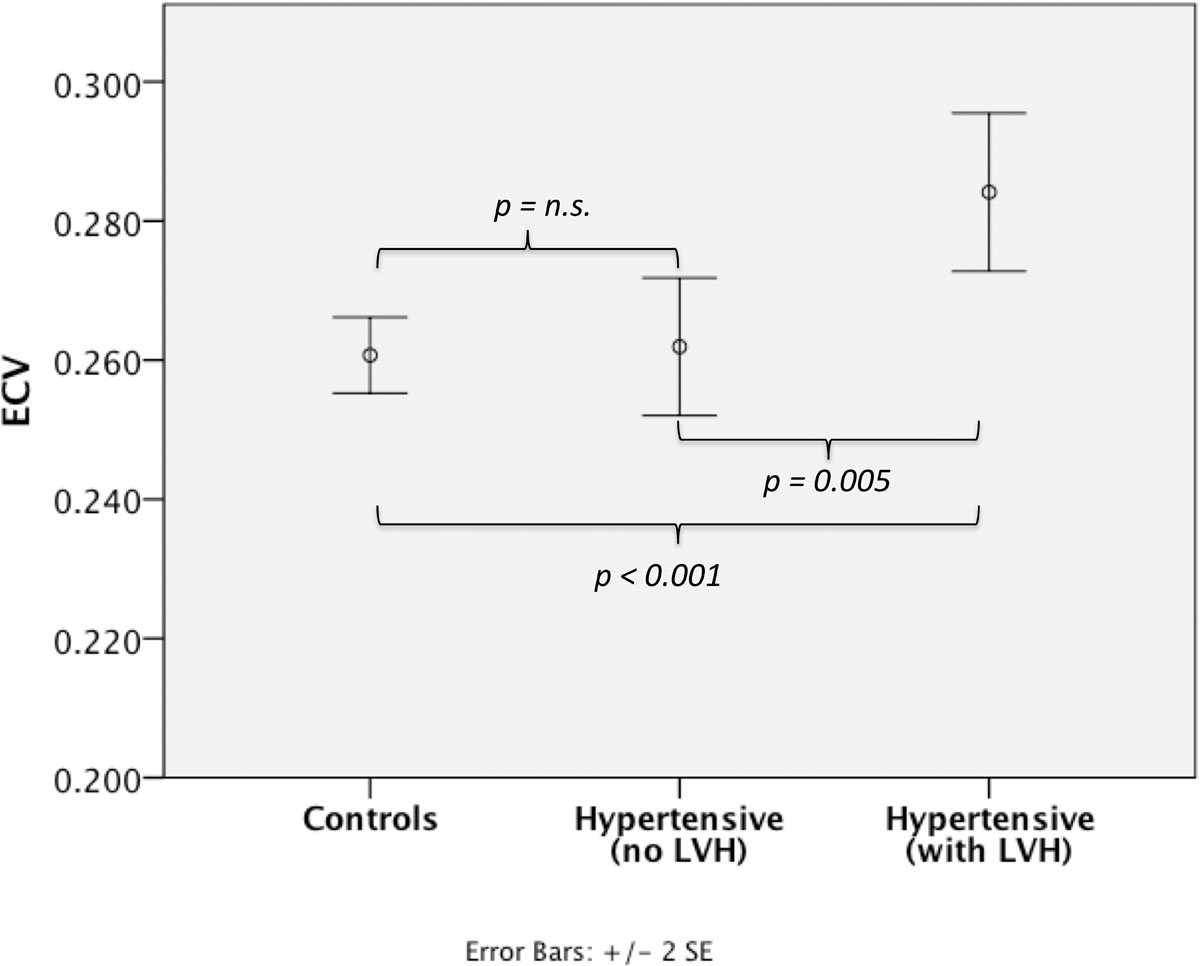
ECV measurements in hypertensives with elevated left ventricular mass index (37%) were significantly higher than those without elevated mass index (0.285 versus 0.262, *p*=*0.005*).

Only 16 hypertensive subjects (37%) had a mass index outside the normal range (male>90g/m^2^; female>78gm^2^ [JCMR 2006, 8, 417-426]), and their ECV was significantly higher than those without elevated mass index (0.285 versus 0.262, *p*=*0.005*; Figure 2). As expected, this sub-group had higher systolic and diastolic blood pressures on ambulatory monitoring (168/96 versus 134/78, *p*<*0.05*), larger end-systolic and diastolic volumes and left atrial areas.

## Conclusions

ECV is significantly higher in subjects with well-controlled isolated, systemic hypertension than in normotensive, healthy volunteers. Elevated ECV was predominantly measured in patients with higher mean blood pressures and hence increased mass index. The overlap between the two cohorts can therefore reflect the success of effective blood pressure control, affecting not only LVH, but also diffuse fibrosis. There was no overall correlation between mass index and ECV, which may suggest that ECV and by inference DMF is an independent factor in systemic hypertension.

## Funding

British Heart Foundation; National Institute for Health Research.

